# Make your own laboratory: A comparative perspective on the logistics and dynamics of DIY biology spaces and communities

**DOI:** 10.1371/journal.pone.0330307

**Published:** 2025-08-25

**Authors:** Anna Verena Eireiner

**Affiliations:** 1 Department of Sociology, University of Cambridge, United Kingdom; 2 Institute of Political Science, University of Tübingen, Germany; University of the Faroe Islands: Frodskaparsetur Foroya, FAROE ISLANDS

## Abstract

Do-It-Yourself (DIY) biology, also known as biohacking or community biology, is a grassroots movement where people conduct biological experiments outside formal institutions. DIY biologists set up their laboratories in garages and community spaces and often acquire their equipment and materials from online marketplaces. While their needs for material resources are comparable to those of academic and industrial laboratories, DIY biologists face greater challenges in acquiring such resources, reflecting the structural disparities between institutional and extra-institutional science. This paper examines how DIY biology spaces are materialized and explores the challenges encountered during this process. Materializing refers to the efforts of DIY biologists to transform their visions of grassroots science into material and immaterial results. Drawing on semi-structured interviews with 23 DIY biologists across Great Britain, Germany, and Canada, alongside observations from online and in-person events, this study highlights how these practitioners position themselves within their respective countries’ life science landscapes. The findings indicate that DIY biologists actively negotiate boundaries between institutional and extra-institutional science, engaging in debates over funding sources, including partnerships with industry, and seeking alternative models of community sustainability. Country-specific innovation and economic ecosystems shape these negotiations. For instance, Canadian DIY biologists embrace entrepreneurial narratives akin to ‘garage start-ups,’ aligning with a neoliberal ethos of innovation. In contrast, their German counterparts gravitate toward the principles of *Mittelstand* entrepreneurship emphasizing stability, regional embeddedness, and responsibility. Despite their diverse approaches, DIY biologists in all three contexts face systemic challenges tied to neoliberal funding structures, particularly a scarcity of financial resources. These insights contribute to understanding how grassroots science adapts to, and resists, broader socio-economic forces, illuminating the dynamics through which science is materialized outside traditional institutions.

## 1 Introduction

DIY (Do-It-Yourself) biologists set up their laboratories in garages, kitchens or community spaces, where they experiment with gene sequencing, custom biosensors and grow glow-in-the-dark plants. The DIY biology movement is thought to have first emerged in the United States in the early 2000s, particularly in large cities such as Boston, San Francisco, and New York [1,2] These hubs of experimentation and technology were home to thriving communities of scientists, both professional and amateur, who sought to democratise access to biotechnology and explore new frontiers in DIY experimentation [3,4]

DIY biology tends to combine libertarian tech-optimism with a DIY ethos, thereby challenging the boundaries of science and scientific expertise. It is generally believed that increasing affordability of biotechnology, the emergence of accessible and simplified standardized procedures tailored for DIY settings, and open-source sharing of knowledge and protocols provided fertile grounds for the formation of the DIY biology movement [5–7].

The global DIY biology movement is driven by three core ideals or principles that shape its identity [8]: First, DIY biology seeks to democratize science by making research infrastructures accessible to all, challenging the divide between experts and the public [9]. Second, the movement is thought to provide a platform for addressing socially relevant scientific questions that are often overlooked by academic research institutions [10,11]. Third, DIY biology emphasizes intellectual freedom, offering an open-ended, curiosity-driven approach to scientific inquiry, free from the constraints of institutional science [12,13].

DIY laboratories are typically set up and populated by individuals with degrees in the natural sciences, i.e., the same group of people one finds in academic, industrial and military laboratories and would thus be considered as biotechnology experts [14]. The results of the 2021 DIY Biology Community Survey support these findings. 92% of the respondents claimed to have at least some college-level education, with 76% either holding or currently studying towards a degree in the natural sciences [8].

DIY biology’s professionalized research communities emerge outside, or ‘extra to’, institutional laboratories, which is why I refer to this type of movement as ‘extra-institutional science’ [15]. Much of the movement is constructed in opposition, or as a better alternative, to traditional research institutions [14]. However, because the community members operate outside the supervision and safety guidelines of established institutions, the movement gives rise to heated controversy around the safety and legitimacy of DIY laboratories [16].

While the movement is internet-native, how the movement is regulated and materialized varies significantly by country. DIY biologists use online platforms to foster a sense of purpose, exchange knowledge, protocols, and ideas [17,18]. DIY biology’s challenge is to strike a balance between a globally networked epistemic community, their countries regulatory environment as well as local communities, practices and identities (see [19] for related work on situated materialization at a local scale).

DIY biologists face varying regulatory environments in Canada, Britain, and Germany. In Canada, the regulatory environment is relatively permissive, with the Public Health Agency of Canada (PHAC) seemingly supporting the DIY biology movement as part of the country’s innovation strategy [20]. In Britain, the Health and Safety Executive (HSE) takes a comparatively more hands-off approach but offers a contact point for DIY biologists to reach out if needed. In Germany, state-level agencies are tasked with overseeing laboratories, including DIY spaces, but because oversight is typically geared toward conventional research institutions, it can be difficult for DIY biologists to find a clear regulatory point of contact. However, at the federal level, the movement has been extensively evaluated in alignment with Germany’s, and the European Union’s, precautionary approach to biotechnology [21].. The precautionary principle advocates for preventive action in the face of uncertain risks, emphasizing safety and regulation even when the likelihood of harm is unclear [22]. Overall, DIY biologists in each country navigate distinct regulatory landscapes when establishing their spaces and communities.

This study explores how DIY biology communities are materialized and which challenges community members encounter in the process. In this context, ‘materializing’ refers to DIY biologists’ efforts of putting their ideas of extra-institutional science into existence, both in material and immaterial ways. Following a co-productionist perspective [23], the analysis examines how scientific and social orders are jointly shaped as DIY biologists build extra-institutional science communities. Adopting a comparative perspective, it investigates the approaches and strategies of DIY biology communities in three different countries: Canada, Great Britain, and Germany. The central research question guiding this study is: How do DIY biology communities in Canada, Great Britain, and Germany materialize their visions of extra-institutional science, and what challenges do they encounter in doing so?

I present and analyse country-specific topics that emerged in conversation with DIY biologists, while keeping with overarching themes that are central to the formation of communities across the countries. Overarching themes include challenges associated with securing funding, forming community spaces, training, and onboarding new members, forming collaborations and networks with other (extra-)institutional scientists as well as DIY biologists’ relationships with regulators, sponsors, and publics. As to better contextualize empirical findings, I give an overview of each country’s DIY biology communities, including their spaces and regional networks.

This comparative investigation reveals how DIY biologists establish their identities and communities within their unique country-contexts. Canadian DIY biologists, influenced by their proximity to the U.S., embrace ‘garage entrepreneurship’ narratives, while Germans align with *Mittelstand* ideals of long-term regional responsibility and sustainability [24]. Country-specific contexts shape DIY biologists’ practices: Canadian regulators build sustained rapport with regulators, British DIY biologists admire U.S. regulatory flexibility, and German groups emphasize the precautionary principle, which is a longtime cornerstone of German biotechnology regulation [22]. Across all three countries, DIY biologists rely on academia for a supply of resources and grapple with challenges such as limited funding, overburdened laboratory community leaders, and inadequate training infrastructures. While many resist industry collaborations to preserve independence, Canadians are more open to such partnerships. Communities materialize globally and locally through hybrid networks, yet resilience varies: German groups sustain activity through online networks, despite disruptions such as the COVID-19 pandemic. On the contrary, their British counterparts saw a decline in memberships after the onset of COVID-19. As DIY biologists balance societal goals and financial pressures in neoliberal systems, they advocate for recognition, clearer regulations, and funding access to solidify their role as legitimate contributors to their countries’ life science landscapes.

This research empirically underscores the idea that DIY biology represents a different modality or approach to conducting science. In traditional scientific settings in academia and industry, the emphasis often lies on formalized institutions, specialized expertises, and standardized methods. In contrast, DIY biology embodies a more decentralized, participatory, and hands-on (DIY) ethos, where individuals and communities become involved in scientific inquiry outside of traditional academic or industry laboratories.

The dynamics and logistics of materializing this alternative science movement show that we cannot only think, but do, science otherwise, i.e., with a different set of social, environmental and economic commitments. Through this examination, I seek to illuminate the potential of DIY biology, showcasing how it offers a pathway towards reimagining and reshaping scientific practices.

## 2 Methods

### 2.1 Country and case selection

This paper comparatively investigates the dynamics and logistics of DIY biology spaces and communities in three countries: Canada, Great Britain, and Germany. All three countries host active DIY biology communities and policymakers have engaged in deliberations and implemented a range of policy actions in recent years. The goal of this research is to better understand how communities in Canada, Great Britain, and Germany envision and materialize DIY biology differently, bridging the tension between a globally networked DIY movement, and country-specific cultures, identities, and socio-economic objectives. The US DIY biology movement has been comparatively extensively studied, with research focusing on its origins, community dynamics, regulatory challenges, and the negotiation between openness and safety in grassroots biotechnology [1,5,7,25]. This focus is partly because existing literature on DIY biology is often rather US-centric, which is why I chose to investigate country-specific materializations of DIY biology in other countries. However, the US DIY biology landscape does feature in this research, as it is an important point of reference for communities in Canada and Great Britain. This may be due to the fact that the US is thought of as the birthplace of DIY biology and hosts many large and well-established community spaces [26], such as Genspace in New York or BioCurious in California, that have shaped early narratives of the movement [27,28]. The relatively permissive U.S. regulatory environment [29] is also frequently cited by international actors as a contrast to their own national contexts.

### 2.2 Data collection

This study draws on 23 semi-structured interviews conducted with DIY biologists: 7 from Great Britain, 8 from Germany, and 8 from Canada. Additionally, email exchanges with DIY biologists provided supplementary insights, and participation in various online and in-person events contributed to the analysis. Interviews were conducted between May 2020 and September 2022, with participants recruited through DIY biology networking events, including the Global Community Biosummit [30,31], as well as referrals from other interviewees (snowballing). Recruitment in community laboratories and events in all three countries also contributed to participant selection.

### 2.3 Sampling and potential biases

The recruitment process may have introduced a selection bias, favoring more extroverted and well-connected individuals, particularly those active in international networks or community laboratories. DIY biologists working independently and/or in home laboratories may be underrepresented in the sample. Participants ranged in age from their early twenties to late fifties, with most concentrated in their late twenties and early thirties. There was a near-equal gender split, and the majority held advanced degrees in the natural sciences, reflecting broader demographic patterns observed in the DIY biology community [29].

### 2.4 Researcher situatedness and reflexivity

This research is informed by the author’s background in Science and Technology Studies, with a particular interest in community-driven and extra-institutional scientific practices. Prior engagement with DIY biology networks, through attendance at community events and familiarity with related literature, shaped the development of research questions and facilitated access to participants. This situated position enabled rapport-building but may also have influenced the framing of interview themes and the interpretation of participants’ narratives. Throughout the research process, efforts were made to reflect critically on how disciplinary commitments and positionality as an academic researcher shaped data collection, analysis, and the representation of DIY biology communities.

### 2.5 Interview design

Semi-structured interviews were chosen to allow for consistency in questioning while providing flexibility to explore specific topics in greater depth. Questions were presented in varying orders depending on the flow of conversation, and participants were encouraged to elaborate freely [32]. Interviews were conducted both remotely via video chat and in person, with all audio recordings made with the prior consent of participants. This approach allowed for rich qualitative data collection, though remote interactions during the COVID-19 pandemic may have influenced participant openness and interaction dynamics. Interviews were conducted in either English or German. For those conducted in German, I personally translated the transcripts into English to ensure consistency in analysis.

Participants were asked about their motivations, challenges, backgrounds in academia and industry, and relationships with regulators and sponsors. Interviews were transcribed using the software Descript and subsequently coded in NVivo. Coding focused on themes such as relationships with traditional research institutions, entrepreneurial aspirations, interactions with regulators and sponsors, and perceived challenges. Thematic codes were refined iteratively to ensure comprehensive analysis.

### 2.6 Ethical considerations

This study is reported in accordance with the Standards for Reporting Qualitative Research (SRQR), including ethical and reflexive considerations relevant to qualitative inquiry. The research has been subject to appropriate ethical review and has been approved by the Ethics Committee at the University of Cambridge, Department of Sociology in January 2020. All participants received a detailed introduction to the project and signed informed consent forms prior to interviews. Anonymity was ensured by replacing personal identifiers with generic descriptors (i.e., “Community Member, Interviewee #8”). Participants were informed about this anonymization process.

### 2.7 Limitations

Data collection was influenced by the constraints of the COVID-19 pandemic, particularly regarding in-person interactions. Virtual interviews may have affected the richness of data compared to face-to-face interviews. It should be acknowledged that the sample includes participants from specific DIY biology communities within each country, which may reflect location-specific perspectives and introduce some within-country variation that is not fully representative of the entire national DIY biology landscape. Additionally, the reliance on events and referrals for participant recruitment may have limited the diversity of perspectives, particularly from less visible or isolated DIY biologists. Nonetheless, this approach provides robust insights into the diverse ways DIY biology communities materialize and interact with their respective regulatory and cultural contexts.

## 3 Canada

Canada’s DIY biology communities operate within various extra-institutional science spaces, from permanent and well-established to pop up laboratories in public spaces. Two well-established spaces can be found in British Columbia, namely the Open Science Network in Vancouver [33] and the Victoria Makerspace [34,35]. Other communities that currently operate without a permanent physical space can be found in Canada’s capital of Ottawa, Ontario [36] and in Montreal, Quebec [37].

Alternatively, some groups make use of community or public spaces for their meetings. According to a founding member, Ottawa’s Biotown DIY biology community often meets online. However, before the pandemic, members of Biotown made use of a public library space to set up their equipment. During the pandemic, the library shut. Thus, they organized themselves remotely. As of October of 2022, the group still runs online community meetings [38]. BricoBio is a Montréal-based community laboratory that indefinitely closed their physical community space in 2022. According to Canadian DIY biologists, Toronto used to be home to a DIY biology community that has seemingly since gone dormant. Furthermore, Canada is home to several university-based community laboratories. These include Pelling Lab at the University of Ottawa, SYNBRIDGE Maker Space at the University of Lethbridge and the Milieux Institute Speculative Life Biolab at Concordia University.

In the following, I detail the themes that emerge in the investigation of Canadian materialisations of DIY biology. First, I give a brief overview over the role of mentorship and the neighbouring US in the making of Canadian DIY biology. Looking at DIY biology spaces, I investigate the topic of home laboratories, which are key to Canadian extra-institutional science. A closely related theme is that of Canadian perceptions of DIY biology entrepreneurship, which seems to invoke narratives of ‘garage entrepreneurship’. I found that Canadian DIY biologists are very aware of how DIY biology activities are portrayed in the media, which is why I added this theme specifically for a Canadian context. Furthermore, I analyse key challenges that DIY biologists face when trying to build their communities, i.e., training new members, and securing funding. Additionally, I detail Canadian DIY biologists’ perceptions of regulation and their relationships with regulators, namely the Public Health Agency of Canada. In doing so, I specifically address their ideals of self-regulation within their communities.

### 3.1 Mentorship & the role of US DIY biology

Canadian DIY biologists benefit from a strong, well-established network of mentorship, with experienced organizers guiding newer community members in building sustainable projects. In this context, ‘sustainability’ refers to the community members’ ability to maintain long-term activity through managing leadership workload, securing funding, providing training, and balancing commercial and communal interests. In interviews, newcomers frequently expressed how invaluable the support of veteran organizers has been in fostering local communities and advancing individual projects.

The Public Health Agency of Canada played an important role in these developments by hosting the Canadian DIY Biology Community Summits in 2016 and 2020. These events served as critical spaces for networking and knowledge-sharing, helping to strengthen the Canadian DIY biology community. Many interviewees noted that these summits provided opportunities to meet like-minded individuals and foster lasting connections.

Canadian DIY biologists often draw inspiration from their US counterparts, viewing the American DIY biology movement as better equipped, more sustainable, and more successful in its endeavours. A notable example comes from one Canadian organizer who described how the two movements — US and Canadian — have developed in tandem. The US’s larger market and availability of equipment have made it a key resource for Canadian communities, with many sourcing laboratory supplies from US pharmaceutical sales or eBay.

As I will explain in the next section, the narrative of ‘garage entrepreneurship’ and the widespread use of home laboratories remain key elements in shaping the Canadian DIY biology movement.

### 3.2 Home laboratories & (garage) entrepreneurship

In Canada, home laboratories are a key part of DIY biology, often integrated into personal spaces like garages. This contrasts with the more reserved attitude towards home experimentation found in Germany and Great Britain, where some DIY biologists even avoid discussing it. Canadian biologists, however, are open about their home labs, often sharing their experiences casually. For example, one biologist described building a home lab with older university equipment and creating a fully functional garage lab by 2010. This openness points to the relatively permissive regulatory environment in Canada, which may make DIY biology more feasible.

The ability to work from home also reflects the economic privilege required for DIY biology, as some biologists, unable to afford lab space, turn to home setups. One community organizer mentioned that if they had access to a house with space for a lab, they might not have needed to start a community lab.

I want to argue that it also ties into the narrative, and positive perception of, the ‘garage entrepreneur’. Canadian DIY biologists specifically emphasise independence from traditional research institutions and entrepreneurship. The ‘credit card and a dream’ narrative is especially pervasive in a Canadian context. Furthermore, ‘garage entrepreneur’ narratives imagine DIY biologists who innovate an entire field from the comfort of their own homes. The mythos of the ‘garage entrepreneur’ might be more present because it holds more currency in Canada, and North America generally, than in Great Britain or Germany.

Hewlett and Packard (HP) is one of the most popular garage entrepreneurship origin stories and is thought to signify the birth of Silicon Valley [39]. The ‘garage entrepreneur’ is thought to signify “innovative ideas, old-fashioned hard work and American ingenuity, bootstrapping resources to chase a dream, a rejection of the status quo, and the freedom of working for oneself” [40, p.6]. These are some of the principles that Canadian DIY biologists value. DIY biologists often use their free time and personal funds to work on their projects. They tend to be rather critical towards neoliberal academia, and, to a lesser degree, towards biotechnology industry corporations [8]. One could argue that although they seem critical of neoliberal academia, they are not opponents of neoliberalism, as self-made entrepreneurship narratives are deeply rooted in capitalist ideology [41].

In fact, Canadian DIY biologists’ belief in the ‘garage entrepreneurship’ narrative present an obvious contradiction in the movement’s identity construction. The ‘garage entrepreneur’ is a quintessential part of neoliberal ideology. In fact, the ‘garage entrepreneur’ or ‘garage belief’ has been criticized: The garage is not as common a locus of entrepreneurship as often believed. Audia & Rider call the garage entrepreneur ‘a contemporary legend that obtains its staying power not from its accuracy but, rather, from its ability to tap common emotions in the portion of the [North] American public that is interested in entrepreneurship’ [40, p.7] Even if garage entrepreneurship is a contemporary legend, it’s a source of inspiration for many Canadian DIY biologists. Entrepreneurship and innovation seem core to the identity construction of the movement. This prioritization of entrepreneurial activities and innovation impacts how Canadian DIY biology communities materialize.

In Canada start-ups seem to be embraced as part of community laboratories. This might be due to the fact that Canadian DIY biologists envision start-ups, and DIY biology entrepreneurship very favourably. They seem to have a complex, at times conflicted, perception of capitalist systems. When I asked one community organizer how they cope with building an alternative epistemic space within a capitalist system, they explain to me that capitalism and DIY biology are not, and do not necessarily have to be, mutually exclusive:

“I think capitalist systems are at a macro level, very damaging, and at a micro level, still fairly empowering. That we’ve had people come through our lab, for instance, with a fairly strong profit motivation, but still able to do their exploratory work in a way that was fundamentally equitable. They shared in the community; it was not damaging.” (Canadian Community Member, Interviewee #22)

Another participant gives a more concrete example of how they perceive innovation and entrepreneurship within a DIY biology lab to function better than within academia. The story involves a group of university students that came to the local community laboratory to develop a piece of laboratory technology:

“The graduate students who came to build their […] robot here -- they did so specifically, because if they had developed that in their supervisor’s lab at the University, they would have owed the intellectual property from that invention or a portion of it to the university, whereas if they came and developed [it] at the makerspace, they do not. And so, it’s this interesting, slightly subversive turning the machinery of the university on itself to let people have their inventions that’s been... that’s been an interesting thing that’s come out of that.” (Canadian Community Member, Interviewee #23)

This story indicates that the Canadian community laboratories do indeed function as alternative epistemic spaces (and an alternative economic space in this case). The graduate students were able to develop their technology outside of the university sphere, hence did not have to give up the intellectual property for their invention. This is to say that at the DIY biology community laboratory, the students are the sole inventors and do not owe intellectual property rights to the space or community. The community organizers seemed pleased that their ‘subversive’ approach was successful. This of course, is grounded in a larger critique of the university, which is thought to limit intellectual freedom and reserves some intellectual property rights of inventions made within its bounds.

It can be argued that historically, traditional research institutions, such as corporations, would control the entire innovation process, from ideation to development, manufacture, market launch, distribution and servicing [42]. In doing so, they maintained strict control over intellectual property rights and re-invested profits in more internal research and development. As the 20th century ended, this ‘closed innovation’ model began to erode due to an increase in knowledge workers, which made it difficult to control the rights to an idea [42]. According to Chesbrough, knowledge workers became more mobile and, paired with the rise of venture capital, new start-up firms emerged that made “efforts to commercialize ideas that have spilled outside the silos of corporate research labs” [42, p.36] The rise of extra-institutional science may indicate that knowledge works have become even more mobile, and thus intellectual property rights even harder to control institutionally.

However, this presents as somewhat of a contradiction. On the one hand, DIY biologists advocate for an open source and open science ethos, on the other hand they are not sternly opposed to traditional intellectual property regimes, as the above example indicates.

While specifically German DIY biologists often voice that they are unsure if DIY biologists should commercialize their inventions and products, Canadian DIY biologists seemed to take a different approach. This is to say that many believe intellectual property rights, and the right to commercialize a product, should lie in the hands of the individual, not with a larger corporation (i.e., a university or industry corporation). This again, seems to be tied to a rather positive vision of the ‘garage entrepreneur’, who, by the power of their own ingenuity, innovates and revolutionizes entire scientific fields.

Canadian DIY biology’s positive visions of entrepreneurship are illustrated by a number of companies in the community biology sphere. I want to briefly introduce two examples: Hyasynth Bio and Amino Labs. Kevin Chen, one of the co-founders of a community laboratory in Montréal is also co-founder and CEO of Hyasynth Bio, a start-up seeking to manufacture “natural cannabinoid compounds without the use of cannabis plants” [43,44]. Kevin and his entrepreneurial activities are favourably mentioned in the Public Health Agency of Canada’s proceedings on the 2016 Canadian DIY Biosummit. Accordingly, the Public Health Agency of Canada states:

“In his presentation, Kevin offered an insight on his educational path which included quitting his Master[sic] Degree Program after 8 months to pursue his entrepreneurial goals. He successfully secured over $800,000 in funding over five years. Kevin also indicated that it cost him $3,000 to start-up his lab, which is significantly lower compared to the cost that is typically required in an academic institution” [45, p.4]

For many years, the community laboratory, and the start-up operated hand in hand. As I mentioned above, the community laboratory has recently ceased operation in 2022.

Another example for Canadian DIY biology-inspired entrepreneurship is the Saskatchewan-based firm Amino Labs. The company and its products really excite some of my interviewees. This is how one Canadian DIY biologists describes the company:

“It’s called Amino Labs. And they have these kits, like it’s called the DNA playground. And it’s mostly made for kids that are hoping to bring them to high school. So high school students can use them in biology classes. And it’s very basic, just like doing bacterial transformations, where you’re making like a lab safe E. coli fluorescent or making it like… create a banana smell or whatever. And so, they have all these kits, and they’re doing it as a business and they’re trying to be… like sell this to the educators.” (Canadian Community Member, Interviewee #1)

In its FAQ section on the company website, Amino Lab offers a definition of the DIY biology movement:

“Do-it-yourself biology (DIY biology, DIY bio) is a growing biotechnological social movement in which individuals, communities, and small organizations study biology and life science using the similar methods as traditional research institutions [sic]. Traditionally, DIY biology is undertaken by individuals with extensive research training from academia or corporations, who then mentor and oversee other DIY biologists with little or no formal training. […] With Amino Labs kits and tutorials, this need for a skilled mentor, which can be hard to find, has been removed; let the kits be your mentors!” [46]

Amino Labs aims to solve a common problem among DIY biology communities, which is a lack of trained mentors to onboard and train newcomers. Amino Labs seems to incorporate DIY biology’s ideal to democratise biology by making spaces and educational resources more accessible. In the definition above, they emphasize that DIY biology is not aiming to change scientific methods but to democratise biology. Interestingly, the company also states that DIY biology is largely populated by trained experts. ’Moreover, the company emphasizes that DIY biology is not only a hobbyist or not-for-profit endeavour but can also include commercial/entrepreneurial activities.

The following is an account of Canadian DIY biologists’ reflections on how their movement is portrayed in the media. This is a theme that almost exclusively emerged in interviews with Canadian community members, which is why it is specifically included in this investigation.

### 3.3 Media portrayals: Biohackers as ‘other’

In recent years, the possibilities of extra-institutional science have increasingly attracted the attention of filmmakers. Canadian DIY biologists often mention two Netflix series, namely the 2020 techno-thriller ‘Biohackers’ [47] and the 2019 documentary ‘Unnatural Selection’ [48]. Both series contributed to launching DIY biology into the public awareness/consciousness and have certainly spurred debate around biotechnological futures.

In ‘Biohackers’ a medical student becomes interested in biohacking and gets drawn into illegal genetic experimentation [49]. The documentary series ‘Unnatural Selection’ centers on genetic engineering, specifically gene-editing technology CRISPR. It portrays home laboratories and self-experimentation [50].

Canadian DIY biologists seem worried about how their movement is portrayed in the media. This is an interesting finding because during interviews, I did not ask DIY biologists about media portrayals, or how they think that publics perceive their movement more generally. Regardless, Canadian DIY biologists regularly brought up this topic on their own.

For one, several interviewees voice concerns and frustration over how popular media depicts their movement. Community members seemed worried that publics would define their movement through the popular media portrayals of alternative epistemic spaces. They seemed specifically worried by the moral and ethical dilemmas around synthetic biology, gene editing and the ownership of genomic data that series such as Unnatural Selection and Biohackers raise. They claim that journalists intentionally chose controversial portrayals of extra-institutional science to appeal to a larger audience.

Moreover, some Canadian DIY biologists do not want to be mixed up with individuals that pursue activities that can be thought of as illicit and dangerous. Several interviewees explain to me that they view DIY biology and biohacking as clearly distinct movements. For instance, they wanted to distance themselves from dangerous public self-improvement projects. In this context, community members would most often bring up a public experiment by US-based Josiah Zayner, who publicly injected himself with a homemade experimental herpes treatment (thereby also injecting himself into the collective consciousness of Canadian DIY biologists). Zayner is also featured on the documentary series Unnatural Selection. Zayner, an aspiring biotech entrepreneur, fabricated a treatment to rid himself of the herpes virus [51]. He declared that if his team “succeed[s] with herpes in even the most minor ways,” they would “move forward immediately with cancer” [52]. However, this project did not come to fruition.

Canadian DIY biologists engage in boundary work when they clarify that the self-improvement culture of biohacking, which they don’t want to associate themselves with, is very different from their community biology efforts. Biohackers are identified as Other by some of my interviewees; they are thought to oppose the values of DIY biology communities. This is to say that individuals like Zayner are thought to act without care for their own and others’ safety. Interestingly, several Canadian DIY biologists are aware of how their movement is perceived in media/film and are concerned about the public’s perceptions of extra-institutional science. These concerns highlight the performative nature of boundary work, where claims to scientific authority are not fixed but continuously negotiated within broader social and institutional contexts [53]. Thus, the boundary work performed by these organizers serves to assert their legitimacy as responsible contributors to scientific knowledge while explicitly recognizing and managing the potential stigma they associate with more controversial biohacking practices,

This, however, is not the only challenge that Canadian DIY biologists face when it comes to materialising their visions. As I will detail in the following, Canadian DIY biologists also experience challenges around training new members and securing funding.

### 3.4 Training new members, funding, & envisioned potentials

Specifically, Canadian organizers mention that it takes a lot of time to train newcomers enough to have them work on more sophisticated research projects. This may be connected to the issue of funding. Most Canadian and British DIY biologists agree that finances present the biggest problem to communities. Like community biology laboratories in other countries, they finance their spaces and activities through a combination of income streams. The most common income streams include membership fees, revenue generated through training and workshops and small grants (e.g., in the (bio-)arts). Canadian DIY biologists argue that the COVID-19 pandemic negatively impacted their membership numbers and thus their revenues. Membership fees, as well as workshop and training revenue were negatively affected by COVID-19 related lab closures.

Overall, Canadian DIY biology community laboratories coped comparatively well throughout the COVID-19 pandemic. Like German and British communities, they were faced with laboratory closures and the need to sustain their spaces with dwindling sources of income. Despite these challenges, many of the country’s community laboratories survived the pandemic and have continued their operations, either in person or remotely. In the summer of 2022, several community organizers report that their membership numbers had started to recover from a decline throughout the pandemic.

Canadian DIY biologists imagine their movement as a way to getting back to how ‘science used to be’. This is to say that they think of DIY biology as a movement that incorporates aspects of 17th century European ‘gentleman science’ [54]. This presents a contradiction to DIY biology’s vision of making science, its practices and spaces, more accessible. ‘Gentleman science’ already conveys that the laboratory was only accessible to males.

In Canadian DIY biologists’ imaginings of their movement, another central contradiction emerges: On the one hand, DIY biologists imagine their tools, facilities, and thus technical and scientific opportunities to be limited. On the other hand, some Canadian community members imagine that DIY biology is more likely than institutional science to foster socially relevant research agendas, innovation, and entrepreneurship. This contradiction may be related to the general techno-optimism that characterizes much of the movement. Moreover, as I mentioned earlier, the prevalent visions of garage entrepreneurship may also mean that DIY biologists perceive their limited capacities less as a disadvantage and more as a prerequisite to a Hewlett-and-Packard like success story.

However, I also found that some Canadian DIY biologists do perceive their lack of resources and their hopes for scientific breakthroughs as a contradiction:

“The truth is like, there is… well at this point, except for some places, there is not too much that DIY biology does. There is some education, there’s some advancements in creating labs and devices and stuff like that. But there’s no, you know, like breakthrough or research or, or anything like that. So, to a certain degree, there’s not too much to show for it. Okay, despite the potential.” (Canadian Community Member, Interviewee #21)

This DIY biologist, like many others, continues to pursue DIY biology because they believe in the movement’s potential. It is noteworthy that the vision of DIY biology’s limited capabilities also plays out in another context: perceptions of risks and regulations. I will address this, among other aspects, in the following section.

### 3.5 Self-regulation & regulator relationships

Canadian DIY biologists have varying perceptions of regulation and their relationships with regulators such as the Public Health Agency of Canada, which is the primary agency in charge of overseeing the country’s DIY biology practitioners. Canadian DIY biologists are vocal about their ideal of self-regulation within their communities. This is in line with the Public Health Agency of Canada’s regulatory approach. Within Canada’s lean biotechnology regulatory environment, the agency provides guidance and networking opportunities for DIY biology communities to cultivate a ‘culture of safety’ that essentially relies on the communities’ self-regulatory capacities [45]. Interviews with Canadian community members indicate that the Public Health Agency’s endeavour of connecting Canadian DIY biologists is indeed successful.

Community members are full of praise for the Public Health Agency’s approach. Canadian DIY biologists give several reasons for why they support a self-regulatory approach. DIY biologists often assert that it would be difficult to do harm in/with DIY biology because members oversee and guide each other. Some community members explain that it is hardly possible to work outside of the communities without that guidance. Accordingly, they argue that there should not be any additional regulatory barriers:

“So, it’s hard to say that there should be more gatekeeping because […] the barriers that already exist to being successful at it are enough that you […] have to be willing to like work with the community. Other people know you and you have to basically […] be part of the community to be successful at it, it’s my impression.” (Canadian Community Member, Interviewee #1)

Moreover, DIY biology is envisioned to have somewhat limited possibilities by both DIY biologists and regulators, which is thought to create an additional layer of safety. DIY biologists hold that corporate and academic labs have better-equipped facilities, which is thought to increase risks of intentional or accidental malpractice:

“At any university, there will be people that make stupid mistakes. And then there’ll be people that do illegal things at universities. I think community labs are really too small to have that happening.“(Canadian Community Member, Interviewee #18)

Furthermore, other community members believe it would be very difficult, and even harmful, to regulate the extra-institutional science spaces and communities because it could ‘stifle’ those who want to pursue science:

“Most […] are just doing very basic kits, basic systems. And then if they ever want to do something more advanced then they usually have to go through a traditional academic, or, you know, business like system anyways, so then, like that regulation […] now applies to that anyway. Right. So, it’s hard to stifle people who just want to do science.” (Canadian Community Member, Interviewee #1)

This interviewee again emphasized that they envision the capabilities of DIY biologists to be limited. Complex scientific work is thought to require the facilities of a traditional scientific institution, which come with a different level of oversight and regulation designed for research laboratories.

Out of the three countries, Canadian DIY biologists perceive regulators most positively. This is not least due to the effort that Canadian regulators have put into building relationships and rapport with community members. The 2016 Canadian DIY Biology Community Summit is an important point of reference when DIY biologists characterize their relationship with regulators. In fact, many members see it as a success in building rapport. One DIY biologist described how they remember the time leading up to the event as well as the gathering itself:

“So, they saw that there was beginning to be a growing DIY bio movement in Canada, and lots of unregulated labs, you know, organized and run by community groups […] often involving, you know, both biologists and artists. And so, they decided they need to get a handle on this. And to, I think, assess how much of a threat it was to public health and safety. So, they held this summit. And I would say that they did a really good job with it, they invited a lot of different DIY bio groups from across the country to present their case studies, basically.” (Canadian Community Member, Interviewee #20)

Moreover, DIY biologists praise the Public Health Agency of Canada for keeping rapport with communities outside of the Canadian DIY Biology Community Summits:

“Health Canada has been fairly proactive and getting in contact with spaces and trying to have these opportunities for discussion.” (Canadian Community Member, Interviewee #22)

Another long-term community member emphasized that they have grown comfortable with their relationship with regulators at the Public Health Agency of Canada. They are of the opinion that regulators trust them, stating that regulators do not put them into the same category as infamous biohacker Josiah Zayner:

“[I am] quite comfortable with the relationship, you know, they obviously, are mandated to have oversight over what happens. That’s not just with community biology, but academic biology, industrial biology, anything like that, where there’s a potential risk to Canadians. You know, they have a mandate to oversee it. And […] they trust us. You know, I think they recognise we’re not Josiah Zayner” (Canadian Community Member, Interviewee #18)

One of the reasons for DIY biologists’ positive perception of regulators is that the Public Health Agency of Canada did not add or tighten regulation (as of early 2023), as this interviewee explains:

“They [Public Health Agency of Canada] did a good job. Just for the fact that they didn’t do... they didn’t add more regulations, which my opinion would have been unnecessary” (Canadian Community Member, Interviewee #21).

However, I want to note that not all community members agree that DIY biology is not risky. A few community members voice that they sometimes worry about publics coming into the lab, who are not necessarily informed about biosafety and security precautions. Nevertheless, when asked if they think that this requires regulatory action, the community members disagree. They held that the risks associated with publics experimenting in community laboratories are still negligible compared to the risks associated with institutional scientific research.

When I asked Canadian DIY biologists if they would like regulators to make any changes to how they regulate and/or support the movement, several state that they do not want any change. However, a few organisers articulate that they would like to be able to have access to research grants or at least discuss funding options with regulators. DIY biologists see community laboratories as a valid alternative to traditional research institutions and are keen to be under similar funding regimes.

While Canadian DIY biologists were at-large content with regulation and community-regulatory relationships, they would sometimes take the opportunity to problematise European biosafety and biosecurity legislation. This is to say that European legislation is sometimes criticised for being too strict and is thus thought to stifle European DIY biology. One community organiser states that they would not want to make any changes to Canada’s regulatory approach to DIY biology. They then used European legislation as a negative example to make their point:

“No, no, I won’t wish for anything. More of the same is exactly what we want. And that from a regulatory perspective, Canada’s approach to DIY bio, and to some extent, the US is a much more fundamentally innovative approach than most other regulatory regimes. […] looking at what some of my colleagues who have gone through […] in Europe has just appalled me. Yeah. And it’s anti-innovative and anti-entrepreneurial and so I think the main thing that I would say to Canadian regulators is just keep it up, keep your hands off, don’t let fear and overregulation destroy the good thing we’ve got going.” (Canadian Community Member, Interviewee #22)

This interviewee’s sentiments echo those of several other Canadian community members. The European Union’s biotechnology legislation is identified as the Other. While Canada’s and the US’ approach to DIY biology is described as innovative, European legislation is thought of as anti-innovative and anti-entrepreneurial.

Having now analysed how Canadian DIY biologists are materializing their communities, I want to turn to the case of British DIY biology.

## 4 Great Britain

Great Britain has seen several DIY biology spaces spring up resulting in community laboratories in many cities such as London, Cambridge, Nottingham, Edinburgh and Manchester. Founded in 2011, London Biohackspace is thought to be the first community-run biology lab in the United Kingdom and even Europe [55]. Some DIY biologists consider DIY biology activity to have peeked around 2016–2017, which one British DIY biologist cited as the “height of the community biology craze” (GB Community Member, Interviewee #12). Since then, some community laboratories have closed their doors. One of these labs is MadLab in Manchester. When asked about the reasons for the lab closure, organisers said that other opportunities unrelated to biology emerged, which is why they decided to take their space into a different direction. A community-biology initiative in Nottingham (OpenGenX) has seemingly ceased operations. Compared to Canada and Germany, few British DIY biology spaces remain. In 2022, DIY biology laboratories in Cambridge and London are the only two community laboratories in the United Kingdom with GM centre notifications, which are formal regulatory approvals required for facilities conducting work with genetically modified organisms [56]. Further north, in Scotland, the ASCUS Lab in Edinburgh was first founded by researchers at the University of Edinburgh. It is a non-profit organisation that aims to be “UK’s largest publicly accessible laboratory for experimentation in art & science” [57,58].

As mentioned above, DIY biology is a highly heterogeneous movement. This section investigates how DIY biologists materialise their spaces and communities within Great Britain. It is, however, not an exhaustive description of the country’s DIY biology movement, its communities, and members. This is to say that specifically the British DIY biology sphere is undergoing changes, some of which are due to the COVID-19 pandemic. I will explore this, and various other challenges, in the following sections.

### 4.1 (A lack of) networks & the US as a point of reference

In my investigation of British DIY biology, I found that the communities share some challenges and experiences with their Canadian and German counterparts. However, what stood out in the context of British communities is that the COVID-19 pandemic hit British community biology spaces especially hard. According to community organizers, memberships in community laboratories dropped dramatically, which not only resulted in a lack of membership fees but also in a lack of volunteers. This, in turn, led to limited organizational capacities. Others experienced rising rents and/or the need to seek out and relocate to a new building.

Compared to Canada and Germany, there are relatively few DIY biology community laboratories in Great Britain. While no British community organizer could name definite reasons for the small number of communities, many hypothesized that high rents, few suitable and affordable properties, and a lack of funding avenues are likely to blame. Others state that they assume that the DIY biology ‘craze’ of earlier years has simply passed. This research explores several likely contributing challenges, many of which are seemingly rooted in the challenges and tensions of organizing an extra-institutional science movement within capitalist systems.

Compared to Germany and Canada, British community biologists seem to operate in relative isolation from one another. When research participants were asked which other British community members they know and are in contact with, almost all state that they do not keep in regular touch with other DIY biologists based in Great Britain. Often, they would actually ask me if I knew of any other British groups and their activity status. Participants could not name any particular reasons for not being in touch with other British groups.

This can be seen as detrimental because the lack of British DIY biology networks may negatively affect knowledge transfer and lobbying towards shared goals, such as DIY biology funding/grants or greater political acceptance. Within Great Britain, DIY biologists do not seem to lend each other the same level of community support that characterises much of the Canadian communities. It is perhaps due to a lack of networking opportunities that cross-country connections among DIY biologists are sparser in Great Britain than in Canada. Canadian DIY biologists often name the Canadian DIY Biology Summits in 2016 and 2020 as a place where they have been able to connect with other community members and organisers. Such an event does not (yet) exist in Great Britain but might be an excellent opportunity to initiate and enhance British DIY biology networks.

However, it is noteworthy that British community members are well connected internationally: interviewees consistently mention their connections to groups in the US, on the African continent, and in various other countries. These connections are often well-established over the course of many years and include project-based collaborations, knowledge transfer and the exchange of prototypes for further testing/validation. Specifically, the number of participants who state that they are connected to DIY biologists in the US was striking. Five out of the seven British participants interviewed named at least one US community that they are in contact with. DIY biologists articulate in various ways that they look at the US and its communities for inspiration and orientation. The US seems to act as a point of reference in terms of regulation, research agendas and when envisioning DIY biology’s place within the life sciences sector.

I mentioned earlier that Great Britain has fewer DIY biology communities than the other countries under investigation. In the following section, I will now address possible factors that contribute to the low number of British community spaces.

### 4.2 The issue of ‘zombie spaces’

One of the main challenges that British DIY biologists report is building sustainable communities. Many DIY biology community organisers talk in-depth about their difficulties of organising and sustaining a local group. The challenges they name include overworked community leaders, lack of financial resources, the provision of adequate training opportunities and striking a balance between commercial ventures and other community members.

A number of British DIY biologists report that it is a great challenge for leadership to balance their workload while building and running their communities. British community organisers/lab leaders are most often individuals with a day job. Community organising is done in their free time and includes not only DIY biology projects but also communication with volunteers, organising events, paperwork and other administrative tasks regarding regulatory compliance, management and maintenance of facilities, equipment and supplies etc. These tasks are described as labour-intensive.

According to British DIY biologists, local communities that are not maintained by the organisers hurt the movement at large. spaces that are not built to run sustainably, i.e., in ways that can be well maintained over time, are sometimes thought of as detrimental. British regulators, as well as those interested in joining a community, would sometimes tell me that their messages to DIY biology communities were left unanswered. This problem is not unique to Great Britain, but stakeholders in the British DIY biology-sphere brought this issue up a lot more often than their Canadian and German counterparts.

Some long-time community members call DIY biology communities that appear active but are largely unresponsive ‘zombie spaces’. In my research, I discovered dozens of unanswered public posts left by newcomers on community laboratory social media channels such as Facebook, Slack and Meetup. These messages are typically queries about upcoming meetings, project support and lab induction events. Community members argue that that these ‘zombie spaces’ stunt the movement’s growth and evolution in two ways: they make it harder for new individuals to join, possibly causing their interest in DIY biology to wane. Additionally, it hinders the exchange with other stakeholders (e.g., regulators, sponsors), thereby making it more difficult for communities to access the resources they need to build sustainable communities (e.g., funding).

Growing the community comes with many challenges. DIY biologists envision the democratisation of biotechnology, but when trying to materialise this vision, training new members poses a big challenge according to British DIY biologists. First, they report that they find it difficult to find enough volunteers to train new members. Second, they voice that the amount of training and individual attention that individuals require to work on a DIY biology project is significant. However, few individuals with the right qualifications volunteer to offer training opportunities. One participant describes this challenge as follows:

“I think having access to really good training is a challenge, partly because by its very nature DIY bio wants to be like almost anti-institutional. And yet a lot of people you’re coming in need significant amounts of onboarding and training and so you have this like you basically need people who have been trained in institutions to run that training and that that’s a challenge to find the people to do it.” (GB Community Member, Interviewee #11)

This statement illustrates the difficulty of bringing scientific knowledge, methods, and training to an extra-institutional setting.

“So I think those are the biggest challenges for that is the mismatch between demand and supply of quality, like training, time and mentorship and support time, which is sort of naturally built in, you know, a lot of learning how to do lab work is almost an apprenticeship-based model, which is built into my academic industrial labs. When you’re running a full time as a full-time job […] you kind of shadow people. You learn how to do it. But I think that that level of input is much more challenging outside a formal context.” (GB Community Member, Interviewee #11)

This is evidence that extra-institutional and institutional scientific spaces allow different kinds of education and training to take place. Accordingly, the interviewee above explains that scientific institutions employ an “apprenticeship-based model” that is hard, if not impossible, to replicate in a community laboratory setting. This indicates that tacit knowledge, which is important in laboratory work, might be harder to gain in a community laboratory. This may be because a community lab may provide fewer opportunities to acquire tacit knowledge than an academic or industry lab.

Instead of replicating an apprenticeship-based model as it is common at institutions, DIY biology laboratories typically run workshops to teach multiple individuals at a time. However, there seems to be a mismatch between the number of individuals interested in learning about biology and the number of trained volunteers who can host such opportunities.

I want to note two contradictions to DIY biologists’ imaginings of, and aspirations for, their movement that were most apparent in the British context. Although DIY biologists are aiming to materialise an extra-institutional science movement, they heavily rely on individuals trained at institutions to onboard new members. Compared to their Canadian and German counterparts, British DIY biologists most often experienced difficulties bridging the gap between experts trained at institutions and transferring this knowledge to an extra-intuitional setting.

Biology as a discipline and its methods and tools are envisioned as easy to grasp for publics/non-experts. However, like the British DIY biologist above, others also say that it takes a lot of individual onboarding and training to enable non-experts to participate in the lab’s project in meaningful ways.

In the following section, I address yet another key challenge that emerges in interviews with British community members: that of finding ways to run communities in financially sustainable ways.

### 4.3 Financial sustainability

Compared to Canadian and German DIY biologists, British DIY biologists spoke about the difficulties of carving out an existence for alternative epistemic spaces within the country’s capitalist system in much detail. British DIY biologists univocally agreed that reaching financial sustainability is the greatest challenge they face in building a DIY biology community. One community organiser detailed their community’s struggles as follows:

“The number one challenge is financial sustainability, if you ask any person who is actually running a DIY biology space. […] Having a business model that works is really top of the list because if you want sustainable income, then you either need to scale or you need to dedicate a lot of space to start-ups, which some labs don’t want to do because it changes the dynamic of the lab. All you need to focus on education and offering paid courses to institutions and schools and other things, and that again, may not match with aspirations of the lab.” (GB Community Member, Interviewee #11)

This interviewee, and other community leaders, shared with me that their goal in setting up a community space was never to establish a business. Specifically, British DIY biologists explained that rising rent prices pose a great problem. They explain that they found it difficult to find a laboratory space that is both suitable and affordable. Communities even had to relocate their laboratories due to being priced out of a rented property.

Moreover, commercial ventures play an important role in the functioning of the community laboratory. Start-up companies and other entrepreneurial actors typically pay a higher membership rate than other community members. Thus, they help to keep the already precariously funded community laboratories afloat.

One British lab organizer states that they often see themselves tasked with using their free time to assist entrepreneurial ventures with their projects, essentially acting as an unpaid assistant. This, however, contradicts the equitable, open science community they envision. Moreover, community leaders mention that commercially oriented groups and individuals are less likely to participate in lab building or training opportunities that would advance the DIY bio community. Instead, they were somewhat single-mindedly focused on bringing their venture to fruition.

Although the DIY biologists I interviewed did not express opposition to paying membership fees, some see these fees as problematic. Membership models somewhat contradict the imaginary of democratizing biology by making it accessible to anyone. One community organizer expresses concern that the membership fee system “does exclude some people [...] by the nature of it.” This is to say that it renders the community member into the consumer of a commodity.

According to the DIY biologists I interviewed, British community laboratories have so far received little corporate funding. In principle, British community organisers voice that they are open to receiving corporate sponsorships to fund their laboratories but did not seem to be eager to pursue this avenue. When asked about this, one organiser clarified that any corporate funding for their space would have to be “wholesome”. Along similar lines, a different organiser explains that sponsors would not and should not be able to interfere in the communities’ goals or agendas. This is interesting, as DIY biologists envision DIY biology as a space of intellectual freedom. This includes freedom from outside interests, such as any vested corporate interest that may come with receiving corporate funding for their communities.

None of the British community leaders considered excluding commercial ventures from their spaces and were in a process of finding better ways of creating cohesion between commercial and non-commercial members. Although some of the British community laboratories have been severely affected by the COVID-19 pandemic, they have shown to be resilient. Some community organisers shared their plans for creating more sustainable communities. These plans include residency models that invite start-ups to donate some of their time to the community laboratory and its members. Others want to distribute the workload to paid, part-time community managers to take pressure of the volunteer leadership. One space is hoping to integrate into a maker space to share the costs and administrative labour. Others want to focus more on organising online, thereby expanding their membership beyond their local community.

In the previous pages, I detailed some of the key challenges that British DIY biologists face, which may help to explain the comparatively small number of communities in the country. At-large, British DIY Biologists seem to be more optimistic about regulation and regulator-community relationship, which are the topics of discussion in the following.

### 4.4 Regulation and a strategic edge in the life science industry

The following investigates how DIY biologists imagine DIY biology-regulatory relationships. In personal interviews and informal conversations with DIY biologists, I asked them about their experiences with, and perception of, the regulators tasked with overseeing and regulating the country’s DIY biology communities. I also asked them if they had any problems with how DIY biology is regulated in Great Britain and if they were hoping and/or advocating for regulatory or political change.

A majority of DIY biologists express that local, regional, or national regulators had never contacted them. They also state that they had not seen a need to reach out to any relevant regulatory body.

In personal interviews, a few British DIY biologists said that they had, in fact, sporadically interacted with regulators. These individuals were all community laboratory organisers. They shared that their experiences with regulators have been positive. Specifically, DIY biologists favourably mention how quickly regulators reply to queries.

Moreover, community organisers express that British regulatory resources are easy to access and understand.

“From my perspective, there is nothing policy related in the UK that has been a barrier to us. […] The UK has amazing resources and documentations. The genetic modification policies like I have not found even in the US [United States of America], and I read a lot of their policies too. It’s nowhere near as detailed as HSE guidance.” (GB Community Member, Interviewee #11)

While most DIY biologists report that they are happy with existing regulatory resources and guidance, others state that the government could do more to clarify how DIY biology fits into existing regulation:

“I guess yeah, altering regulation, if that’s, that’s what the government feels, if the direction they’re going in, would definitely make things easier for DIY biology spaces. And kind of, I guess, making it clear where DIY biology fits within that.” (GB Community Member, Interviewee #15)

While DIY biologists most often said that they are generally content with regulatory interaction (or a lack thereof) and the available biosecurity and biosafety resources, they took issue in other respects. This is to say that a majority of British DIY biologists who participated in this research voice discontent over what they perceive as a lack of political recognition and support for their movement and its communities. British DIY biologists argue that they are motivated and skilled to address pressing problem but see themselves limited by a lack of resources. A number of interviewees thought this issue can be solved by greater political backing of the movement.

More specifically, when asked about the political change they would like to see, British DIY biologists imagined their movement within national strategies for life sciences education and innovation. One DIY biologist explains that they envision DIY biology communities to get funded for the education that they are able/aim to deliver:

“I think looking at the role of informal learning and lifelong learning for adults for biotechnology […] is kind of a strategic thrust of the government. I think that like building on that, there’s a lot of policymakers particularly in the UK and the US and other countries that have a strong life sciences sector, you know a part of the life sciences capacities almost always involves skilled workforces. And I think that’s one of the ways in which they could think about DIY bio spaces or supporting access to bio facilities and training in a kind of adult education context is thinking about skilled workforce.” (GB Community Member, Interviewee #11)

This statement indicates that DIY biologists imagine their extra-institutional science movement to compliment the country’s life sciences sector. In a similar vein, this DIY biologist imagined the movement within the British innovation landscape:

“I like to keep up with high level thinking about how DIY bio can fit into, like, the government’s drive to make biotech and life sciences in the UK a really, really strong industry... and there’s much more that they can do.” (GB Community Member, Interviewee #11)

In the minds of many DIY biologists, extra-institutional science does not only fit the life sciences and biotechnology industry but is imagined as giving the country a strategic edge. Practically speaking, DIY biologists voice that support from the government would be most useful in the form of grants and other financial resources.

Between 2012 and 2018, various UK governmental bodies issued reports on emerging biotechnologies, including synthetic and DIY biology [59–61]. The Health and Safety Executive (HSE) is the primary agency responsible for overseeing DIY biology. However, UK regulators have taken a cautious, “wait-and-see” approach. HSE does not maintain direct communication with DIY biologists and instead relies on intelligence from external sources, such as academics studying DIY biology. One HSE official acknowledged that while DIY biology is not currently seen as a pressing concern, the agency is monitoring developments to ensure they can respond if needed.

Brexit has prompted regulators to reconsider how biotechnology is currently governed. Some DIY biologists speculated about post-Brexit regulatory changes that might result in more permissive biosafety and -security regulations. Indeed, government officials have been consulting with some DIY biologists on the topic of post-Brexit regulations. One community organiser mentions that a government official had visited their laboratory to this end:

“We had a visit from the Health and Safety Executive. They wanted to talk to us about policy making for DIY bio in the Brexit context. They were reviewing a lot of GM [genetic modification] legislation, so they wanted to come and basically consider if they needed to consider DIY bio, like is this something that should be on their radar? […] So yeah, one of their policy people came and visited for the evening and we showed her around and kind of talked about what we do. You know, she left. I mean, not scared.” (GB Community Member, Interviewee #11)

However, an official at HSE confirmed that there are no plans following Brexit to make any regulatory changes that would pertain to DIY biology.

Some DIY biologists wishfully spoke of the comparatively less restrictive US-regulatory environment, specifically the permissive genetic modification regulation. In the eyes of some DIY biologists the comparatively strict biotechnology regulations in Great Britain are at odds with DIY biologists’ ideal to make biotechnological research more accessible. One research participant articulate that they imagine DIY biology’s main advantage to be the greater accessibility of biotechnology research. However, compared to the US, they consider the country to fall short in making biotechnological research more accessible:

“The difference in the UK compared to the US is that I know some people in the US have home labs to get started. […] here you couldn’t even do that if you wanted to because of the GM [genetic modification] regulations. So, it’s even harder to access a space where you could do some biotech experiments. And almost all biotechnology recombinant protein expression involves having genetically modified organisms. And you cannot do that outside of a lab that has a GM center registration. So, I mean, the barriers are very, very, very high.” (GB Community Member, Interviewee #11)

This, again, shows that the US is an important point of reference for British DIY biologists.

## 5 Germany

Similar to the other countries of investigation, DIY biology laboratories can often be found in larger university cities. For instance, in 2018 bio.kitchen, a DIY biology community laboratory under the umbrella of the Technical University of Munich, opened its doors [62]. In western Germany, there is Lab3, which is community space in the city of Dortmund [63] (Lab3, 2023). However, most of Germany’s DIY biology infrastructure is located in Berlin and its greater region. There is biopunk.kitchen in Potsdam [64]. BioLab Eberswalde, which has since closed and moved some of its equipment to Potsdam [65]. More central to the capital of Berlin, there is Top Lab [66]. Additionally, there is Art Laboratory Berlin, a space with a focus on bioarts [67].

In my research on German DIY biology, I found a comparatively large amount of community organizing to be done online. Online forums and group are a key component of how German DIY biology communities are materialised, i.e., organized, grown and sustained. Like in the other two countries, there are some German DIY biology communities that have a presence online, but it is not apparent whether the community is still active or has gone defunct.

Having already pointed out some of the key differences and similarities in the ways that Canadian and British DIY biologists materialise their movement, the case of German DIY biology shows yet another set of country-specific themes/particularities in the materialisation of DIY biology. Compared to their counterparts in Canada and Great Britain, German DIY biologists seem highly diverse in their identities and roles within the already heterogeneous realm that is DIY biology. They also seem very flexible in defining and building extra-institutional science spaces, often utilizing online forums and groups to build their communities and coordinate their practices. Furthermore, German DIY biologists seem to work with a wide repertoire of funding opportunities that they creatively locate and make use of. Their perception of funding and a lack of community-regulator relationships presents more of a challenge, as I will show in the following.

Before doing so, I give an overview over how communities are organized, specifically emphasizing the importance of online forums and global networks among German community members.

### 5.1 Online organizing and global networks

In comparison to how Canadian and British DIY biology communities are materialised, much of the German community organizing and collaboration seem to happen online. It is striking how many online forums, community platforms and instant-messenger groups German DIY biologists have formed. Many of these online groups and forums focus on a specific topic, such as the cultivation of mushrooms or sustainable agriculture. Community members tend to be active in a whole range of these groups and forums.

The forums and groups also aid in organizing in-person meetups and workshops. Germany’s community members will often meet up in public spaces, such as parks and pubs. Because so much of the community organizing is done online, the lack of community laboratories seems less of an issue than in the other countries under investigation. This might be one of the reasons why German DIY biology communities seem to have fared comparatively well throughout COVID-19 and various lockdowns.

German DIY biologists are well-connected among each other but also internationally. German interviewees had the most international ties as compared to community members in Great Britain and Canada. Several interviewees talked about traveling within Europe and even all the way to South America to collaborate on DIY biology projects. Moreover, they attend international camps to learn new techniques, work on projects and meet collaborators. Again, many of these international collaborations are formed and sustained through online communities, which further points to the importance of online spaces in the organization of German DIY biology.

However, German DIY biologists not only use a variety of (online) spaces to organize, but they are also comparatively heterogeneous in the makeup of their communities, which I will analyse in more detail in the following.

### 5.2 Diversity of DIY biology identities and spaces

The DIY biology movement is not only heterogeneous in terms of practices and themes, individuals practicing DIY biology also often wear many different hats. I found this to be especially true for the German DIY biology community. In their identities, or roles, they make up a comparatively heterogeneous community. This is to say that community members would often simultaneously identify as community organizer and environmental activist, artist, animal breeder, mycology or ornithology enthusiast, academic researcher, computer hacker, entrepreneur and so on.

As I mentioned earlier, one of these roles would often introduce members to DIY biology. Some DIY biologists mention to me that they practiced what can be defined as DIY biology for many years before first learning about the term DIY biology and the movement more generally. Many of the German community members explain that through their other engagements, they came across the movement more or less by chance.

German community members seem to combine their different identities with relative ease. The result are various DIY biology community circles and forums that are tasked with a range of different, sometimes very specific topics. Some of the biggest groups in the German DIY biology community are tasked with sustainable agriculture and food systems, mycology, hardware and alternative medicine These forums, or groups, often overlap in their organization, membership, and activities. For instance, ideas, queries, and projects from the sustainable agriculture groups would be carried to DIY biology mycology circles and vice-versa.

This exchange seems to help German DIY biology by attracting new members and offering various activities and themes. This diversity is likely, at least in part, grounded in German DIY biologists’ success in setting up and establishing lively online forums/platforms. It takes comparatively few resources to run an online forum vs. a physical community laboratory.

German DIY biologists also seem flexible in setting up their alternative epistemic spaces. Alternative epistemic spaces can take many forms, such as a community member’s basement, a meet-up at a public park or pub, an online forum or a (community) garden.

German DIY biologists tend to be mobile and often work in distributed projects. They are connected with other DIY biology enthusiasts online and will travel to different corners of the world to do hands-on project work on-site for days, weeks or even months on end. While German DIY biologists experience many of the same difficulties in establishing, and keeping up, permanent community laboratories, they have found ways to successfully sustain and grow local communities. Their need to make-do with semi-permanent, temporary or virtual community spaces may be interlinked with Germany’s comparatively strict regulatory environment. As the regulatory hurdles to open a laboratory are comparatively high, some communities may not have the resources (financial) or otherwise to set up a permanent laboratory space.

However, German DIY biology continues to grow and DIY biologists are not only creative in materializing spaces, but they are also equally creative in securing funding opportunities. In the following, I will detail their approach to funding, and some of the challenges that German DIY biologists face.

### 5.3 Micro-grants & challenges

Like their Canadian and British counterparts, German community members acknowledge that challenging the expert-public divide proves to be strenuous in practice. One of the challenges to make the space accessible while also providing guidance to new members:

“When you have your own laboratory, you cannot just let people in and say ‘okay, just go ahead’. You have to take [new members] by the hand. There are very few autodidacts out there. Most people are used to having things spelled out in workshops or other formats. It costs a lot of time and effort to guide people. (German Community Member, Interviewee #3)

This is a sentiment that is shared among community organizers in all three countries under investigation. Accordingly, several German community organizers talked about how they can only onboard non-experts by volunteering as mentors, which takes a lot of time and resources. While this system of mentorship is putting a strain on many British organizers, their German counterparts appear more optimistic. One community member deems this system of volunteer mentors to be successful for their DIY biology community and even Germany as a whole:

“The people that are not matriculated at a university can still join a community lab. These are places that are run by volunteers. This is what really sets volunteering in Germany apart, people do it because they see why what they do is meaningful.” (German Community Member, Interviewee #3)

However, like in other countries, German DIY biology is still mainly populated by individuals that hold degrees in the natural sciences and could thus be considered ‘experts’.

Moreover, German DIY biology ‒ like British and Canadian counterparts ‒ does not operate in isolation from the academy. Instead, German DIY biology communities depend on universities to source their equipment and even strike up cooperations with university members. One community member explains that even though they have no personal or academic connection to the universities in their city, they still experience the entanglements between academy and extra-institutional science:

“So, I think they’re interwoven. I mean, our group at [name of community lab] has been entangled with university […] For instance, […] getting equipment that the university doesn’t need anymore, like we got this giant flow hood that they were going to get rid of, because it had a broken fan. But then we were able to, I think, repair the fan or get a new one. […] And clearly, some of the people at [name of community lab] also are doing workshops with the university. And so, it’s kind of like a spectrum of connections. (German Community Member, Interviewee #9)

The ’spectrum of connections’ that the community member mentions suggests that extra-institutional science and the academy do not operate independently from each other. In carving out their own communities, extra-institutional scientists seem to depend on institutional science in major ways, e.g., for collaboration and equipment.

In sourcing funding for their communities and projects, DIY biologists depend on established institutions in more ways. Securing funding for their extra-institutional spaces and activities is one of the key challenges for DIY biologists. While German DIY biologists also list funding as one of their concerns, specifically amidst rising rent prices in major cities, finances seem less of an issue than for communities in Great Britain and Canada.

Much of the German DIY biology organizing is done online and utilizes public spaces or semi-permanent laboratory setups, which helps to keep costs low. This is to say that they often do not have to cover the costs of permanent community laboratories, e.g., rent and utilities.

Even more importantly, German DIY biologists seem to be skilled at securing micro-financing opportunities for their projects. One German community organizer explains how they approach the challenge of finding funding:

“I think one has to be creative. Once you have concept, you just have to do some research […] into which funding scheme fits. This is because if you are working on a concept that is not intended for [product] development, you just have to think what the [project’s] societal benefit is. You have to think about who will reward that. And, I think, money is always available. One has to understand that that is not the problem. There is only a lack of concepts and people that are brave enough to get started, I would say.” (German Community Member Interviewee #3).

According to German community members, funding opportunities come in the shape of collaborations with small companies, awards and grants by charitable organizations, academic outreach programs and science competitions. Some community members are also able to secure funding via JOGL, a DIY biology community platform that offers grants to community projects [68]. All of these different sources of funding are usually micro-grants valued between a few hundred and a few thousand Euros.

German DIY biologists also seem creative in tailoring their ideas and project to existing avenues of funding. Several interviewees talked about either their successes or their future plans to secure grants targeted at artistic projects and explorations.

“I want to move more into the direction of arts, because I have a feeling that arts funding is easier to get than funding in the field of technology […]. Maybe one can also tap into the category of citizen science […] I know [of individuals] who were able to get funding relatively easy. Right. It is definitely a field of exploration. I don’t have that much experience yet, but I know that certain things are simply possible. I think, generally speaking, it is easier to get funding in Germany” (German Community Member, Interviewee #7).

These two interviewees’ statements above stand in stark contrast to the numerous funding challenges that other community members report.

### 5.4 Commercialization and entrepreneurship: Making Mittelstand

In Canadian DIY biology, the “garage entrepreneur” narrative drives the movement, emphasizing self-reliance and innovation. In contrast, German DIY biologists view entrepreneurship through a more cautious lens.

The topic of commercialization and entrepreneurship is comparatively contested among German DIY biologists. There are competing visions over how, and if, extra-institutional science is compatible with for-profit, commercial activities. More specifically, German DIY biologists do not seem to agree whether for-profit activities can be reconciled with visions of socially relevant research goals and intellectual freedom.

This is to say that I find German DIY biologists are at-large comparatively critical of industry corporations and their for-profit targets. DIY biologists would sometimes find themselves pursued by industry actors, such as management consultancy firms, venture capitalists or large corporations. German community members seem to think of these advances as a bit peculiar, at times even insidious. Like their British counterparts, they often voice that they would not compromise their visions of what DIY biology communities, and their projects should strive for. While not eager to enter into, or even pursue, industry cooperations, not all German DIY biologists generally condemn community members that cooperate with industry:

“I noticed in the DIY bio community at large, there’s a little bit of a funny thing that like, it’s kind of trending right now. And you can get funding through certain type of corporate stuff. And there’s even like a strangely capitalist influence on it at times too. So, it’s just something I noticed. I don’t think it’s bad. Like I support people in all their projects.“(German Community Member, Interviewee #9)

For some DIY biologists, their projects aim to contribute to socially relevant areas of research; entrepreneurship is neither their goal nor an intended side-effect. However, DIY biologists want to share their results and products with the world. Finding the right manner to reach others beyond the community can come as a challenge.

This is illustrated by a story that a German community member shared with me. The community member collaborated with others to sustainably grow edible mushrooms. Their project was largely self-funded. Soon, their techniques proved to be very successful, resulting in sizable mushroom harvests. The community member explained that growing the mushrooms was the initial challenge they were focused on. However, once successful, they struggled with sharing their harvests with others. The aspects of sales and distribution also proved to be laborious. Thus, turning their rather successful DIY biology project into more than a hobbyist project came with challenges that they were neither equipped nor too eager to handle.

In my research, I encountered several of these self-funded, collaborative approaches to projects that can potentially benefit individuals beyond the community. Their approaches bear resemblance to the ‘garage entrepreneurship’ ideals that are prevalent in Canadian DIY biology. This is to say that they most often ‘bootstrap’ their resources, work independently and rejected the status quo [40, p.6].

However, German community members’ (attempted) materialisations of ‘good’ or ‘desirable’ entrepreneurship differed crucially. I found that when attempting to materialise entrepreneurship, German DIY biology community members imagine German ‘Mittelstand’ rather than Silicon Valley-inspired entrepreneurship.

In Germany, ‘Mittelstand’ is a term used to refer to small to medium-sized enterprises, which are often family-owned [69]. German ‘Mittelstand’ is thought to embody certain values such as continuity and long-term focus, strong regional ties, continuous investment into the workforce, innovativeness, financial and social responsibility [42,70].

German Mittelstand is associated with slow, long-term growth, while Silicon Valley entrepreneurship is associated with rapid growth. However, German Mittelstand and Silicon Valley startups are not an antithesis. Furthermore, German Mittelstand can be thought of as “competitive, innovative as well as growth oriented; sometimes by other means that are less visible than the well-known uni- or decacorns from the Silicon Valley” [69].

One German community laboratory specifically sets out to bring about Mittelstand entrepreneurship. Even after their initial funding has ceased, community projects are offered a place to bring their project (and business) to fruition. A community organiser explains how the making of ‘Mittelstand’ within an extra-institutional science space works:

“Our [community lab organizer] is really trying to work with start-ups and to help create spin-offs. This is done so that people can have an income […]. At universities, you always get these initial projects. You get initial funding from the university and then people are either dropped or they are caught by the big vultures, which means that they go to business angels and venture capitalists and all of that. But to have a regional catchment basin, where one says, ok, here you have some more room to further develop yourself and your business idea. It did not exist in this manner. This is our [lab organiser’s] vision to give people the opportunity to start a business without selling out to the big guys” (German Community Member, Interviewee #4).

As the statement above indicates, instead of imagining the community lab as a continuation of the ‘garage entrepreneurship’ narrative, some German community members imagine DIY biology laboratories as ‘safe spaces’ from the venture capitalists and Angel investors that are typically associated with Silicon Valley entrepreneurship.

“This start-up thing shouldn’t go like ‘get that product ready until it gets bought, or you get buy-outs’ but instead one should follow through long-term: until you can build real Mittelstand. This is a vision that I find really cool“(German Community Member, Interviewee #4).

The community ideals of building Mittelstand emphasise visions of entrepreneurship that value sustainable growth, responsibility, and longevity. Instead of working with industry, DIY biologists are envisioned to receive a safe space within community laboratories to follow through on their journey to a Mittelstand company.

This again, is evidence that DIY biology is co-produced with country-specific vision of what desirable entrepreneurship and innovation looks like. There is no way one of envisioning and materialising extra-institutional science, instead extra-institutional science is co-produced with country-specific visions of desirable futures in a multitude of ways. Like in for the previous cases, I now want to address aspects of how DIY biology is regulated in Germany, specifically how community members perceive regulation and regulators.

### 5.5 Regulation and a (lack of) community-regulator relationships

This section investigates how German DIY biologists imagine DIY biology-regulatory relationships. In personal interviews, I spoke to community members about their experiences with, and perceptions of, regulators and regulations. Additionally, I asked them if they were hoping or advocating for regulatory or political change.

In my analysis of Canadian materialisations of DIY biology, I explain how some Canadian DIY biologists criticize how European countries regulate DIY biology. Specifically European Union regulation is viewed as too strict and even used as a negative example. However, I found that when asked about their perceptions, German DIY biologists were at-large content with how their activities are regulated. Canadian DIY biologists sometimes argue that DIY biologists in Europe cannot perform genetic modification, which is thought to stifle innovation. German DIY biologists did almost exclusively not share this point of view. Instead, several interviewees argue that genetic modification is not needed in their DIY biology activities:

“It [regulation] is restricted in comparison [to North America]. Genetic modifications is a small area. My projects, if I manage to find time and funding, […] it has nothing to do with genetic modification. There is so much more that you can do than just engineering bacteria.” (German Community Member, Interviewee #2).

The vast majority of interviewees explain that they are content with regulation on genetic modification. Moreover, German community members most often articulate that they agree with Germany’s, and by extension the European Union’s, relatively strict biotechnology regulatory regime.

Activities conducted in biological laboratories are categorized in containment or biosafety levels. Depending on the containment level, different oversight regiments, precautions and regulatory restrictions apply. Containment levels range from 1 through 4, with 1 indicating the lowest and 4 indicating organisms that pose the greatest risk to humans and the environment. All three countries make use of this international classification system [59,71,72, p. 16].

German community organizers seemed overall content with operating S1 laboratories, which are considered the lowest biosafety level laboratories. They held that upgrading to higher security laboratories is not necessary for their communities and their projects and is therefore not part of their future plan for their extra-institutional scientific spaces.

Instead, DIY biologists see their movement as a valid part of the German science and innovation landscape. Accordingly, they imagine contributing scientifically, e.g., by providing laboratory infrastructure to support public health in times of crisis. Examples include administering and processing tests for the public during the global Covid-19 pandemic and helping to supply medication.

I found that specifically German DIY biologists reflect a lot on the security and safety implications of their movement. German interviewees would often give me long lists of potentially dangerous scenarios that could hypothetically arise at an extra-institutional laboratory. During interviews, they would sometimes take the time to explain how a rogue individual could utilize such a laboratory to build a bioweapon. Other popular scenarios include laboratory accidents, e.g., situations where pathogens escape the laboratory.

German DIY biologists seemed to have these scenarios, even if unlikely, on their minds and would often explain how each scenario could be avoided and/or prepared for. Overall, German DIY biologists did not only seem to have well-researched security and safety aspects but also argue in favour of clarifying regulatory standards for extra-institutional laboratory spaces.

German DIY biologists’ very well thought out (often hypothetical) biosafety and ‒security scenarios seem to be guided by the precautionary principle. The precautionary principle implies that in a situation of uncertainty, the presumed risk is interpreted as if it was factual [22, p. 15]. Not only does the precautionary principle guide the German regulatory approach to DIY biology but it also seems to be a guiding principle in the visions and materialisations of the German DIY biology community.

Earlier, I explain that German DIY biologists are flexible in finding ways to work without a physical community lab space. Indeed, community members are keen to make-do with temporary set-ups such as in co-working spaces, basements, and gardens.

However, German DIY biologists sometimes articulate that Germany’s comparatively strict biosecurity and biosafety regime necessitates this ‘minimalist’ approach to DIY biology. Like this German DIY biologist explains, this is due to the many regulations and the high cost of complying with these regulatory requirements:

“The word laboratory is associated with many regulatory requirements. […] We are in a niche with DIY biology. A lot of activity happens at home. But when you try to create spaces, then actually one has to… it happens really quickly that thousands of regulatory requirements arise. One cannot comply with those because it is really cost intensive. […] Of course it would be fantastic, if regulation, the whole thing, could be made simpler.” (German Community Member, Interviewee #3).

This sentiment is shared by a number of German DIY biologists. This is especially true for those who organize community spaces and events and thus have frequent touch points with the regulatory aspects of DIY biology. However, it has to be noted that German DIY biologists are not generally opposed to regulation, in fact, they seem rather keen to adhere to existing regulation. It is primarily the cost of equipping a laboratory space according to regulatory requirement that is problematized.

German DIY biologists report that they either have sporadic or no contact with regulators. Interestingly, a number of German DIY biologists express that they see it as their responsibility to network with policymakers and to let them know about their movement and its goals. This community member has long-term networking and even hand-on activities in mind to familiarize policymakers with DIY biology:

“Maybe we just need to get in touch with regulators […] I met a few public officials once but, I think […] one has to make a point to stay in touch and invite individuals to events. Maybe even invite them to join in [our activities], so that they can simply see what this is about. They need a concept for what is happening. They [regulators] can’t truly understand otherwise […]. They think this [DIY biology] means some experiments in a basement but can’t comprehend what and who is involved.” (German Community Member, Interviewee #3)

Additionally, German community organizers often seem optimistic that, if they made an effort to reach out, regulators would be more accepting of DIY biology:

“There are amazing [DIY biology] communities that are working on projects and are tearing down barriers. Many community members are incredibly engaged people. But I think first they [policymakers] have to understand that. I think once they’ve grasped that, have really experienced it, then politicians and policymakers, they’ll be able to relate. I think we, as a community, need to be more proactive in reaching out to them.” (German Community Member, Interviewee #6)

The thought of not having done enough to reach out to policymakers would in some cases make DIY biologists thoughtful. A few even asked me who I think they should contact. In fact, German DIY biologists sometimes seemed eager to learn more about regulatory competencies in their state and even on the federal level:

“Do you know someone? Have you been in contact with someone at a ministry, or someone who is tasked with biosecurity or something similar? I wouldn’t know who to contact. Someone? Anyone?” (German Community Member, Interviewee #7)

Interviewees seem quite unsure what the correct contact person is and whether to look at agencies at a regional, state, or federal level. This, of course, provides evidence that DIY biologists would like to communicate with policymakers. Regulators should make an effort to build bridges between themselves and DIY biology communities, e.g., by organizing events for networking or communicating who the responsible contact people on a regulatory level.

Like their counterparts in Canada and Great Britain, they hope for additional funding opportunities and other resources, such as equipment and (laboratory) spaces. Some DIY biologists imagine that they would ideally be included in funding competitions that are available to institutional researchers. They also express that they are hoping that DIY biology’s visibility will increase, which is thought to also increase the public’s and regulators appreciation for the movement.

Moreover, German DIY biologists express that it can be difficult to find biosecurity and safety relevant regulation. Community members argue that it is in everyone’s best interest that regulators specify if and how regulation applies to DIY biology.

## 6 Discussion

This study of DIY biology communities in Germany, Canada, and Great Britain highlights the diverse ways grassroots scientists materialize their communities and spaces while navigating unique national contexts.

While Canadian DIY biologists critique EU regulations broadly, they may overlook the nuanced differences among member states. For example, Germany’s precautionary principle-based approach to regulation emphasizes safety and hypothetical risk mitigation, aligning well with the values of many German DIY biologists. In contrast, British DIY biologists benefited from comparatively lenient EU legislation before Brexit, which they often juxtapose with the more permissive U.S. regulatory landscape.

DIY biologists negotiate their position between institutional and extra-institutional science. While community labs aim to democratize science, they remain heavily reliant on academia for resources, including second-hand equipment and expertise. Across all three countries, DIY biologists aspire to reduce this dependency. Industry collaboration, however, presents a more contentious issue. British and German DIY biologists express reluctance toward industry sponsorship, fearing it could compromise their independence and intellectual freedom. Conversely, Canadian DIY biologists are more open to such partnerships, viewing them as a practical means to secure funding and achieve research goals.

Country-specific innovation models further shape how DIY biology communities define themselves and materialize their spaces. Canadian DIY biologists often embrace “garage entrepreneurship,” rooted in neoliberal values of self-reliance and innovation, and frequently align with startup culture. German DIY biologists, on the other hand, draw inspiration from the country-specific innovation model Mittelstand, which emphasizes long-term stability, regional ties, and social responsibility [24]. These differing visions reflect broader national economic and cultural frameworks, illustrating how global DIY biology movements are co-produced with local contexts. British DIY biologists take a broader approach, situating their efforts within national strategies for life science education and innovation, emphasizing their role as a complementary to traditional institutions.

The materialization of DIY biology communities takes diverse forms, from online networks and occasional meetups to neighborhood community labs. German DIY biologists demonstrate the resilience of hybrid models, maintaining activity through online engagement and temporary spaces, even during disruptions like the COVID-19 pandemic. In contrast, British DIY biology communities struggled to sustain themselves during the pandemic, reflecting differing levels of organizational stability. Additionally, country-specific networks and experienced members play crucial roles in building cohesive, sustainable communities. Canadian DIY biologists benefit from long-standing mentors who guide newcomers, while German community members often collaborate across multiple groups to strengthen their networks. British DIY biologists, however, report challenges in establishing long-term collaborations, contributing to the decline of some groups.

Training and education remain significant challenges for DIY biology, revealing contradictions between the movement’s goals and realities. While DIY biologists aim to democratize science and make it accessible to non-experts, they often struggle to provide adequate training. The apprenticeship-style learning common in academia is difficult to replicate in community labs due to a lack of resources and mentors. As a result, DIY biologists are exploring alternative methods to convey tacit knowledge, including the use of educational kits and community-developed training standards. These efforts aim to make scientific education more inclusive while addressing the logistical challenges of operating outside traditional infrastructures.

Regulatory relationships further shape the trajectory of DIY biology. Canadian DIY biologists generally have positive interactions with regulators, who have taken steps to build rapport and support the movement. In Britain, regulators are accessible but less actively engaged, while German DIY biologists often lack clear points of contact and express a desire for stronger relationships with regulatory agencies. Despite these differences, DIY biologists in all three countries express general satisfaction with their regulatory environments, though they consistently advocate for greater access to funding, clearer guidelines, and increased political recognition.

Finally, the neoliberal systems in which DIY biology operates present both opportunities and constraints. Community organizers often face the dual pressures of sustaining their spaces financially and resisting the commodification of their values and practices. While Canadian DIY biologists more readily embrace industry partnerships, many British and German practitioners are wary of the influence of commercial interests. Nonetheless, all DIY biology communities must navigate the challenges of funding, leadership burnout, and training deficits as they work to establish themselves as legitimate contributors to their countries’ life science landscapes.

This study shows that DIY biology is a global movement shaped by local contexts, balancing the ideals of accessibility and democratization with the practical realities of resource constraints and regulatory environments. By examining the materialization of these communities in Germany, Canada, and Great Britain, this study illustrates the diverse ways in which extra-institutional science spaces adapt to and reflect the values, challenges, and opportunities of their specific country contexts. More than just a comparative account, this research highlights the potential of DIY biology to reimagine scientific practice by offering alternative, socially embedded, and collaborative ways of doing science.

## Supporting information

S1 File Checklist supplement. (DOCX)
